# Risk of musculoskeletal disorders in pepper cultivation workers

**DOI:** 10.17179/excli2021-3853

**Published:** 2021-06-07

**Authors:** Marta Gómez-Galán, Ángel-Jesús Callejón-Ferre, Manuel Díaz-Pérez, Ángel Carreño-Ortega, Alejandro López-Martínez

**Affiliations:** 1 CIMEDES Research Center (CeiA3), Department of Engineering, University of Almería, Ctra. Sacramento, s/n La Cañada, 04120 Almería, Spain; 2 Laboratory-Observatory of Andalusian Working Conditions in the Agricultural Sector (LASA), Avda. Albert Einstein, 4. Isla de la Cartuja, 41092 Seville, Spain; 3 CIAMBITAL Research Center (CeiA3), Department of Engineering, University of Almería, Ctra. Sacramento, s/n La Cañada, 04120 Almería, Spain

**Keywords:** musculoskeletal disorders, greenhouse, ergonomics, health and safety, biomechanics

## Abstract

Agricultural workers have an increased risk of musculoskeletal disorders, mainly due to the manual nature of the work. This study assesses the level of physical well-being in pepper cultivation workers in Almería (Spain). The objective was to analyze pepper cultivation tasks performed in the Almería-type greenhouse, using the OWAS (Ovako Working Posture Assessment System) and RULA (Rapid Upper Limb Assessment) methods. The OWAS results showed a normal posture percentage of 53 %, a medium risk of 30 %, a high risk of 16 %, and a very high risk of 1 %. The body areas most affected were the back and legs. The RULA assessment found high risk/action levels, with 50 % of the postures corresponding to level 3, 35 % to level 4, and 15 % to level 2. Improvements are therefore proposed; these include: redesigning tasks, mechanization, training, team development, and improving the workers' physical condition. The OWAS and RULA data may have overestimated the results, as workers do not appear to be limited in performing tasks and do not normally request sick leave.

## Introduction

Musculoskeletal disorders (MSD) are one of the most important occupational illnesses worldwide (Enez and Nalbantoglu, 2019[13]). In Europe and Spain, the most frequent reasons for sick leave are due to such disorders (INSST, 2012[32]). They result in higher labor costs for companies, workers and for states (EU-OSHA, 2007[15]).

In the agricultural sector, most of the tasks are manual, which places a great physical burden on the employees (Vanderschilden, 1989[62]). The consequences are clear - commonly occurring musculoskeletal disorders in agricultural workers (Riemer and Bechar, 2016[53]). Agricultural mechanization lowers the percentage of MSDs, but even so, manual labor is unavoidable (Fathallah, 2010[18]).

In the prevention of agricultural risks, musculoskeletal disorders are a priority, along with psychosocial risks, prevention management, the study of respiratory and dermatological diseases, and chemical exposure. No one type of risk is more important than another so their prioritization will depend on the authorities (Fenske et al., 2002[19]).

One feature of the agricultural sector is the association between musculoskeletal disorders and a poor safety climate (Arcury et al., 2012[3]); in contrast, a climate in which good safety practices prevail, with good occupational health and safety management, favors a greater work capacity and worker commitment (Perkiö-Mäkelä and Hirvonen, 2019[50]).

Musculoskeletal disorders tend to increase with age, with lower educational levels and in the presence of other diseases (Hoy et al., 2018[28]; Perkiö-Mäkelä and Hirvonen, 2019[50]). In agricultural workers, they are generalized in nature (although they predominantly occur in the lower back; Sejari et al., 2014[58]), but these workers do not usually seek medical attention. Musculoskeletal disorders are underestimated, which suggests that sick leave due to this condition is higher than that recorded by the authorities (Holmberg et al., 2002[27]).

Preventive medicine and health promotion are often weak points in the agricultural sector. For this reason, occupational risk prevention programs must be adapted to the geographical, legislative, and population characteristics, as well as to the cultivation systems and the types of tasks (Schenker, 1996[57]); other authors even suggest taking into account the influence of present-day climate change on working conditions (droughts, floods, heat waves, and cold snaps, etc.; Belcore et al., 2020[6]).

Workers need training, information and awareness regarding preventive practices to improve their working conditions (Imeah et al., 2020[30]; Vyas, 2012[63]) accompanied by a good health surveillance system and prevention plans (Luque et al., 2012[42]). Incorrectly carrying out agricultural tasks and not following the prevention plan recommendations favor musculoskeletal disorders (Pistolesi and Lazzerini, 2020[52]).

The prior ergonomic design of the workplace will minimize musculoskeletal risks (Koiri, 2020[39]). In addition, crop mechanization will reduce workplace accidents (Narimoto et al., 2020[45]); however, mechanization is associated with musculoskeletal problems derived from vibrations, which are usually minimal due to improvements that have been made (Benos et al., 2020[7]). Furthermore, it is associated with adopting forced postures as a consequence of handling the machinery (operating the gear lever, command levers, brakes, clutch pedal and steering, along with postures taken when looking, observing and manoeuvring; Romano et al., 2020[54]).

Another innovative option would be to use exoskeletons for agricultural tasks, especially for the back and knees. Their main drawback would be adapting them to different cultivation conditions and different gradients with the falls that could result (Upasani et al., 2019[61]).

It is more difficult for small farms to mechanize due to the costs involved, whereas this is not the case for larger agricultural concerns. Consequently, more musculoskeletal problems occur on small farms (Imeah et al., 2020[30]). However, the costs of mechanization and implementing preventive measures hinder their widespread adoption, especially in developing countries (Karsh et al., 2013[35]).

Repetitive arm and hand movements are the most demanding actions undertaken by agricultural workers in Spain (67 %). MSDs are also observed in the neck (23 %) and lower back (50 %) (Almodóvar-Molina et al., 2012[2]; Esteban-Buedo et al., 2013[14]). Furthermore, few MSD studies have been conducted in agriculture (Nguyen et al., 2018[46]) even though it is a sector where numerous risks exist (Son et al., 2010[59]).

Methods have been developed that allow one to assess musculoskeletal disorders. These are divided into direct methods (using sensors), semi-direct methods (observation and assessment software) and indirect methods (questionnaires). Semi-direct methods are classified according to three factors that lead to the appearance of MSDs: forced postures, repetitive movements, and manual load handling (Gómez-Galán et al., 2017[23]).

Examples of assessment methods include: OWAS (Ovako Working Posture Assessment System; Karhu et al., 1977[34]), REBA (Rapid Entire Body Assessment; Hignett and McAtamney, 2000[26]), RULA (Rapid Upper Limb Assessment; McAtamney and Corlett, 1993[43]), the Standardized Nordic Questionnaire (Kuorinka et al., 1987[40]), and the Quick Exposure Check (David et al., 2008[11]).

The present study aims to assess the physical well-being level of pepper crop workers in Almería (Spain). Pepper cultivation tasks carried out in the Almería-type greenhouse have been analyzed using the OWAS and RULA methods.

## Materials and Methods

### Greenhouse description

**Figure 1:**
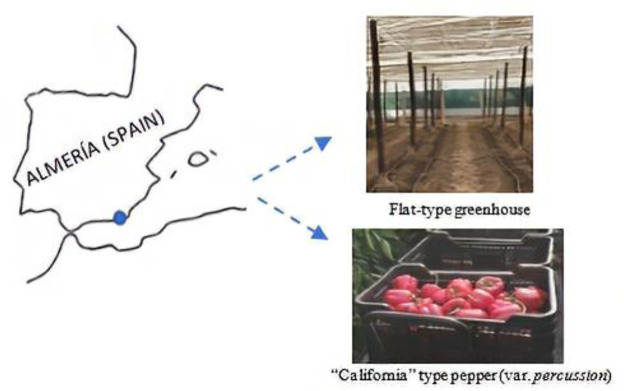
Location, greenhouse and crop

The greenhouse is situated in Almería province (Spain). It has a total surface area of 2,000 m^2^ with sandy soil and drip irrigation. It is a flat-roof, Almería-type greenhouse intended for the cultivation of “California” pepper (var. *percussion*) (Figure 1(Fig. 1)). The crop growing period was approximately 7 months. Three thousand plants were transplanted.

The number of workers varied according to the task performed. The minimum was one and the maximum were seven working simultaneously on the same job. All the workers were men above the age of consent.

This study focuses on assessing the postures assigned to each task, not on the workers who perform them. The postures adopted by the pepper crop agricultural workers in Almería-type greenhouses are very similar. Therefore, the workers' characteristics are not considered, rather the differentiated postures assumed during the cultivation process. The study sample is the number of postures.

**Figure 2:**
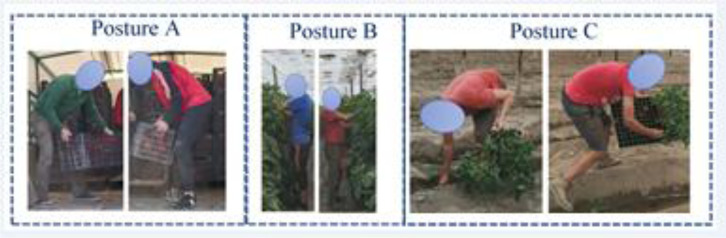
Similar postures for the same tasks but carried out by different workers

In Figure 2(Fig. 2), three tasks are presented, each performed by two different workers. One can see that the postures are very similar despite the person who adopts them.

### Assessment methods used

To select the assessment method, a decision matrix has been constructed (Table 1(Tab. 1); References in Table 1: Colombini, 1998[9]; Corlett et al., 1979[10]; García et al., 1997[20]; Hignett and McAtamney, 2000[26]; Karhu et al., 1977[34]; Kemmlert, 1995[36]; Kilbom et al., 1986[37]; Kuorinka et al., 1987[40]; McAtamney and Corlett, 1993[43]; NIOSH, 1981[47]). Each method has been rated from 1 to 4.

Two semi-direct observation methods, OWAS and RULA, have been applied in this study.

The OWAS method was developed originally for the steel industry in Finland. It is a method for assessing forced postures. OWAS allows one to identify up to 252 different postures. To do this, it establishes four different positions for the back, three for the arms, seven for the legs, and three load bearing intervals (Karhu et al., 1977[34]; Takala et al., 2010[60]).

The RULA method is based on assessing repetitive tasks. RULA analyzes the position adopted, considering the arms, wrists, forearms, trunk, neck and legs. It focuses on the upper extremities. It also takes into account the repetition frequency of the posture or if the posture remains static. Finally, it considers the load (McAtamney and Corlett, 1993[43]; Takala et al., 2010[60]).

The most important differences between OWAS and RULA are shown in Table 2(Tab. 2) (References in Table 2: Karhu et al., 1977[34]; McAtamney and Corlett, 1993[43]; Takala et al., 2010[60]).

In both methods, the work observation can be done directly or by taking videos or photographs. After selecting and assessing the observations using the two methods, levels of risk (OWAS) or action (RULA) are obtained. Four levels are differentiated in both cases, the fourth being the most harmful. According to the levels obtained, corrective actions will be required (Karhu et al., 1977[34]; McAtamney and Corlett, 1993[43]; Takala et al., 2010[60]).

OWAS allows the risk category of each posture to be obtained using a prior coding. This consists of a 4-digit code (one for each area of the body and the last one for the load; Appendix A). In addition, it assigns a risk level to the posture adopted by each part of the body, which depends on its repetition percentage (INERMAP, 2011[31]; Karhu et al., 1977[34]).

RULA obtains the action levels using scores. The arm, forearm, wrist and wrist gyration are included in Group A. The trunk, neck and legs correspond to Group B. Scores are obtained for both groups and these are modified (scores C and D) by taking into account the load and repetition frequency, or static posture. From these scores, a total score is obtained (between 1 and 7 points) that will be included in an action level (INERMAP, 2011[31]; McAtamney and Corlett, 1993[43]).

These methods have been used in numerous fields of knowledge for the ergonomic analysis of workers. They should not be applied individually but together with other methods to provide more comprehensive results (Gómez-Galán et al., 2017[23], 2020[21]).

### Application of OWAS and RULA

**Figure 3:**
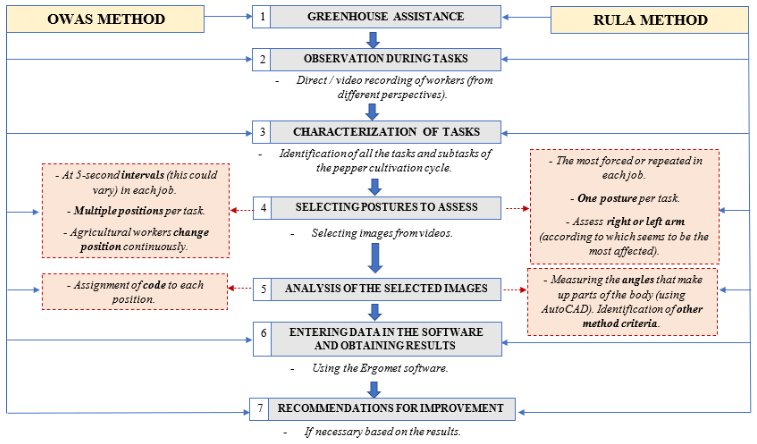
Procedure for applying the OWAS and RULA methods

To apply the methods, the following procedure was performed (Figure 3(Fig. 3)) based on the elements from both methods; these can be consulted in the original articles (Karhu et al., 1977[34]; McAtamney and Corlett, 1993[43]):

### Camera and software

The equipment used was as follows:

- A Nikon COOLPIX S210 digital camera was used for video recording during the observation period. It takes images of 8.0 million effective pixels. Digital zoom: up to 4x. Optical zoom: 3x.
- The angle measurements from the images selected to apply RULA were performed using AutoCAD computer-aided design software.
- The application of the OWAS and RULA methods was carried out with the help of Ergomet software (INERMAP, 2011[31]).

### Tasks identified

During the observation period, the tasks carried out in the pepper cultivation process were identified (Appendix B). Greenhouse maintenance tasks were not taken into account. Several tasks and subtasks were differentiated. These are described below, and each is assigned a code that will be used in the Results section.

Task 1: Transplantation

- Making holes: the initial cultivation subtask consisting of making holes in the sand with the help of a cultivator hoe (T1).
- Planting: inserting the pepper plant with its root ball into each hole made (T2).

Task 2: Laying horizontal strings

Horizontal strings are placed from one end of each growing line to the other to support the plants. This task was carried out five times during the cultivation period. The postures adopted varied depending on the plant height. The two occasions considered to be the most harmful a priori were assessed, namely, the first (when the plants had hardly grown at all) and the fifth (when the plants were already at a considerable height).

- This was divided into two subtasks:
- Tying the horizontal strings: tying the strings at one end of the growing line (T3: short plants and T5: tall plants).
- Extending the horizontal strings to the other end: the worker extends the string to the other end of the growing line and ties it off (T4: short plants and T6: tall plants).

Task 3: Placing the vertical ties

- Placing the vertical ties: placing the vertical strings, fastening them at one end to the horizontal strings, and at the other, to the upper wires suspended inside the greenhouse. This task was performed twice with very similar postures, one of which was assessed (T7).

Task 4: Phytosanitary treatments

- Phytosanitary treatments: manual application of phytosanitary products. Using a hose connected to a tractor with a tank.
- This task was performed several times during the cultivation period. Only one of these was assessed since it was noted that the plant growth was not sufficient to modify the workers' posture in this subtask (T8).

Task 5: Preparing the crop

- Placing the drip irrigation lines: adjusting the irrigation lines in the sand for them to work correctly and to avoid tripping while carrying out tasks. Two workers laid the drip irrigation lines, each pulling from one end (T9).
- Laying down plastic: placing plastic in each crop line to avoid weeds growing and humidity rising to the roof (T10).

Task 6: Staple Placement

- Staple Placement: Staples are placed between the horizontal strings and the ties to ensure fastening. This was carried out on four occasions, for two of which the postures were assessed (shorter and taller plants; T11: short plants and T12: tall plants).

Task 7: Introducing auxiliary fauna against pests

- Introducing auxiliary fauna: The worker tips beneficial insects from a pot onto some plants randomly (T13).

Task 8: Tying strings to pillars

- Tying strings to the pillars: to prevent the plants from overturning, other strings are used to tie the previous strings to the greenhouse pillars (T14).

Task 9: Harvesting

- Picking the peppers: Cutting the peppers, sometimes with the help of sharp tools. Collecting the peppers in boxes on trolleys. This was done on four occasions yet only two of them were assessed because they were practically the same postures (T15: first harvest and T16: last harvest).
- Loading: The collected pepper boxes are loaded onto a lorry for transportation. This was only assessed once as the same postures were always adopted (T17).

Task 10: Cleaning

- Removing the plants and carrying them to the lorry: pulling up the plants and removing them from the greenhouse once the cultivation is over. These are piled onto carts and then loaded onto a lorry (T18).
- Removing the strings: all the strings in the greenhouse are cut down and collected (T19).
- Sweeping: when the crop has been removed, the greenhouse is swept to complete the cleaning (T20).

In total, 20 subtasks were analyzed using the OWAS and RULA methods.

## Results

### Results with OWAS

A total of 1,000 postures adopted by pepper cultivation workers were assessed. Specifically, 50 postures were selected for each subtask.

#### Risk levels by subtasks

**Figure 4:**
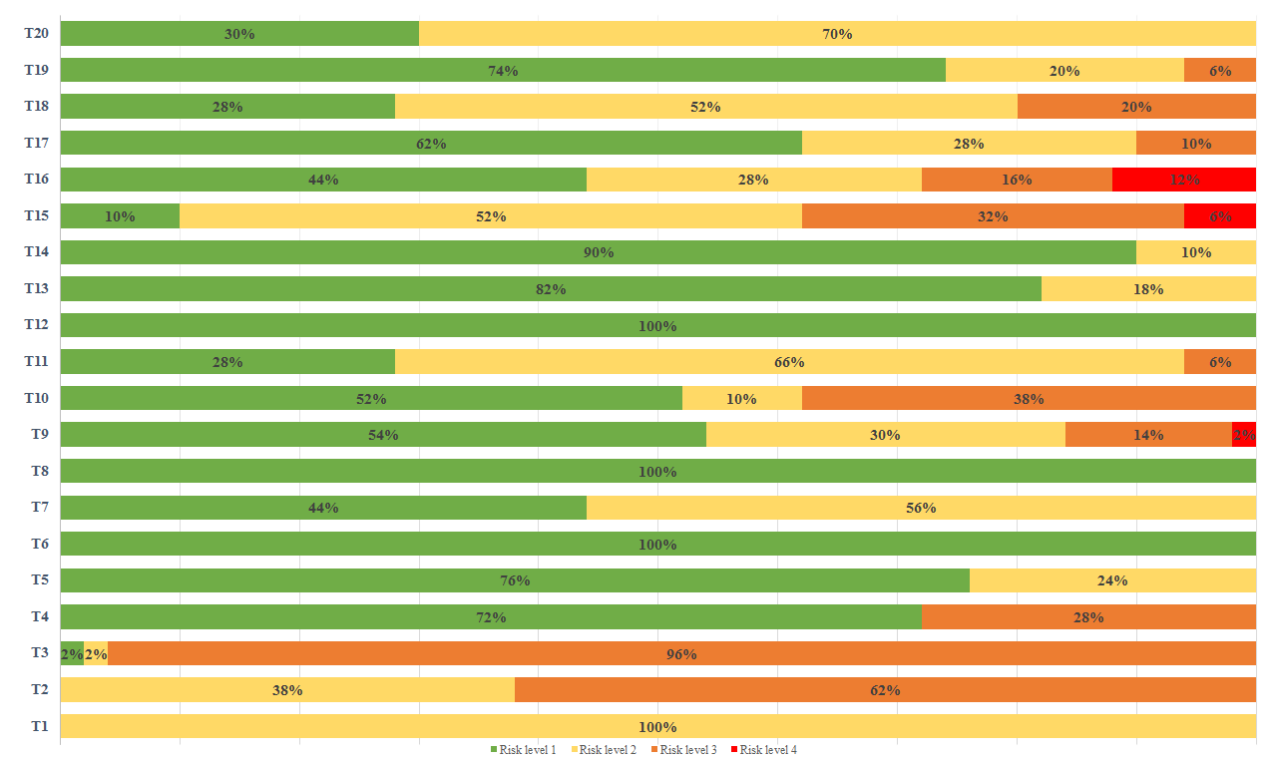
Risk levels in each subtask

Figure 4(Fig. 4) shows the differentiated levels of risk in each subtask and the percentage of postures corresponding to each of them.

According to Figure 4(Fig. 4), risk level 1 is the most common (it appears in 18 of the 20 subtasks). It predominates in “carrying horizontal strings to the other end (tall plants)” (T6), “phytosanitary treatments” (T8) and “staple placement (tall plants)” (T12) encompassing 100 % of the postures.

 Risk level 2 is the second most presented in the subtasks (16). “Making holes” (T1) stands out, in which 100 % of the postures correspond to this level.

The higher levels are presented to a lesser extent. Level 3 appears in 11 of the 20 tasks. It presents a fairly high percentage in "horizontal string tying (short plants)" with 96 %. Level 4 only appears in 3 tasks with very low percentages.

Only 3 subtasks are classified in the four risk levels.

### Risk levels by postures and subtasks

Next, the different postures in each subtask and their risk level are presented (Appendices A and C). The percentage of repetition when adopting each of them is shown in Table 3(Tab. 3).

Of the 20 tasks analyzed, “carrying horizontal strings to the other end (short plants)” (T4) and “phytosanitary treatments” (T8) are the ones that adopt the least variety of postures, only 3 (Table 3(Tab. 3)). In both, the most repeated is 1171 (straight back, arms down, walking, and a load of less than 10 kg), and this is predominately a low risk (level 1). Its repetition rate is 72 % in T4 and 74 % in T8.

Conversely, the highest number of different postures occurs in “pepper picking (the last harvest)” (T16) with a total of 18. The highest repetition percentage (18 %) corresponds to code 1161 (straight back, arms down, on one's knees and a load of less than 10 kg), with a level 1 risk.

There are 5 postures that are most harmful (risk level 4), corresponding to 3 tasks. None of them coincide, their codes being: 4151, 3361, 4141, 4261 and 4161. What they all have in common is that the supported load is less than 10 kg and that the back is bent and turned, except for one, in which the back is only turned.

There are 24 other postures that are also unfavorable for the worker (level 3). These are carried out in 10 different tasks. Code 2151 stands out (bent back, arms down, unbalanced bent legs, and a load of less than 10 kg) in "horizontal string tying (low plants)" (T3) with a repetition of 84 %.

The remaining postures are included in risk levels 1 and 2, the majority of which are considered normal postures (level 1). “Making holes” (T1) stands out as all of its postures belong to risk category 2, with 2131 being repeated more frequently (back bent, arms down, one leg extended and the other bent, and a load of less than 10 kg).

#### Risk levels by body areas and subtasks

OWAS also allows risk levels to be categorized according to the number of times each body posture is adopted. The results are shown in Table 4(Tab. 4).

The predominant back positions during cultivation are the straight or bent back. The highest repetition percentages for both are presented in each task. “Sweeping” (T20) is the exception, in which the back is bent and turned (Table 4(Tab. 4)).

The arms are down in most of the postures adopted and this is not harmful to the worker (risk level 1). A higher risk occurs when the worker has both arms raised during the two subtasks included in the placement of horizontal strings (T5 and T6). In both, the repetition percentage is somewhat higher than 30 %.

The highest risk levels appear for the back and legs. The most unfavorable posture that the worker performs is bending the legs, but with the weight unbalanced between them, during the “tying horizontal strings: low plants” subtask (T3). This is because this forced posture is performed in 84 % of the leg positions adopted. For legs, all four risk levels can be seen, although level 1 predominates.

Lastly, the load is always less than 10 kg, except for “load” (T17), in which loads between 10 and 20 kg are also supported.

### RULA results

There was a total of 20 images analyzed with RULA. This method assesses independent postures, not sequences. Only one posture is assessed for each task, the one considered a priori to be the most forced or repeated (see section “Materials and Methods”).

Table 5(Tab. 5) presents the angles obtained for each part of the body, as established by RULA (Appendix C).

Using Ergomet software (INERMAP, 2011[31]), the following results were obtained (Table 6(Tab. 6)).

Table 6(Tab. 6) indicates which part of the body (right or left) was assessed for each posture. In addition, it presents the scores obtained and the corresponding action level.

The highest C score of those obtained (6 points) is presented in the “planting” subtask (T2). The highest D score was for “load” (T17) with 10 points.

The total score established by RULA ranges between 1 and 7 points. In Table 6(Tab. 6), one can observe that all the scores have high values, with none below 4. The maximum score is obtained for 7 of the 20 positions analyzed, resulting in an action level of 4. The predominant action level is 3, which appears in 10 tasks. Only 3 tasks are characterized as lower risk, these being “phytosanitary treatments” (T8), “staple placement: tall plants” (T12) and “introducing auxiliary fauna” (T13) with the lowest score (4) and an action level of two. No subtask is considered acceptable.

### Results summary for the cultivation as a whole (OWAS and RULA)

**Figure 5:**
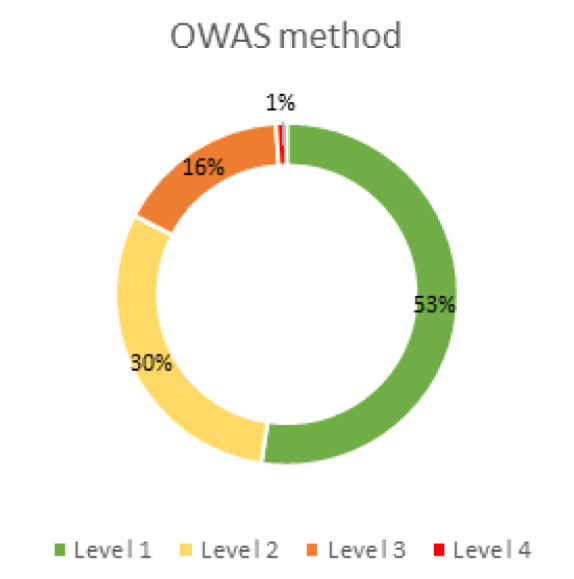
Risk levels in pepper cultivation according to the OWAS method

**Figure 6:**
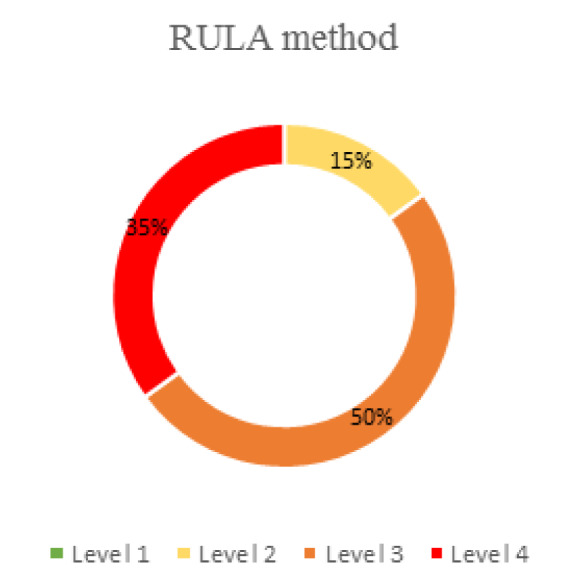
Action levels in pepper cultivation according to the RULA method

A summary is presented of the risk/action levels obtained for the pepper cultivation process as a whole, according to each of the methods (Figures 5(Fig. 5) and 6(Fig. 6)).

For the results obtained using OWAS, the lowest risk levels (1 and 2) stand out with percentages of 53 % and 30 %, respectively (Figure 5(Fig. 5)). However, with RULA, the opposite is the case. The levels with the highest percentages (35 % and 50 %) are the most harmful (3 and 4) (Figure 6(Fig. 6)).

## Discussion

**Figure 7:**
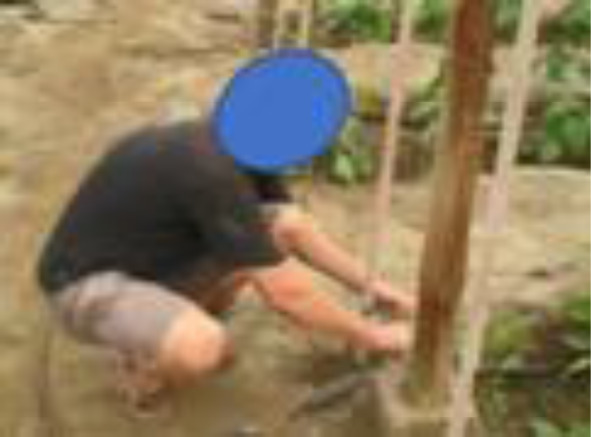
Subtask T3

According to the OWAS results, one of the tasks presenting a greater number of postures (96 %) with a high risk (level 3) is “horizontal string tying: short plants” (T3) (Figure 4(Fig. 4)). This is detrimental to the agricultural worker's musculoskeletal system. Corrective actions should be taken straight away. This is probably due to the fact that the plants were still short when the subtask was carried out. The worker would have to adopt forced postures to reach down almost to ground level (Figure 7(Fig. 7)). This is verified by the results which show that, in most of the postures, the worker had his back bent and legs bent, and was unbalanced (Table 4(Tab. 4)).

Tasks performed at ground level usually require more awkward postures. This coincides with an assessment of pineapple farm workers using OWAS. The authors concluded that adopting postures such as bending down, kneeling or squatting frequently led to the appearance of musculoskeletal disorders, mainly in the back and legs (Salleh et al., 2019[56]). In T3, this is also true (Table 4(Tab. 4)), since the bent back and bent legs present risk levels of 3 and 4, respectively. These must be corrected soon (level 3) or urgently (level 4).

**Figure 8:**
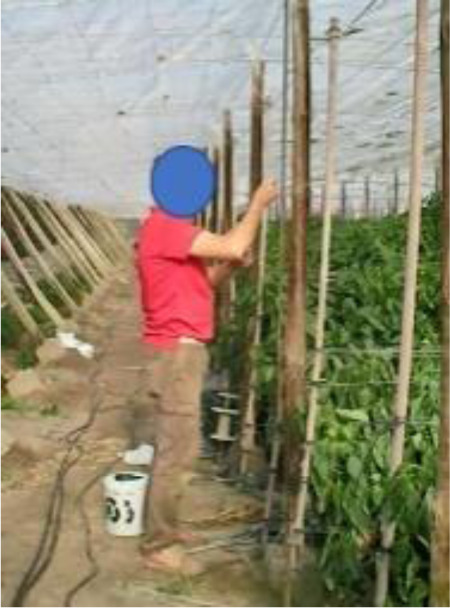
Subtask T5

The same task was assessed on the last occasion it was carried out during the cultivation period (T5). By this point, the plants had grown (Figure 8(Fig. 8)). Here, the opposite happened, with 76 % of the postures being classified as normal, and not requiring correction. The remaining percentage belonged to a level 2 risk, which does require corrective actions but in the near future (Figure 4(Fig. 4)). This is because the back and legs were straight in most postures, thanks to the height of the plants. However, the arms were more affected than in the previous case, as they had to be raised repeatedly to carry out the task (Table 4(Tab. 4)).

The same was also demonstrated in a study of agricultural workers in Sweden. Performing the tasks in a standing or walking posture decreased back discomfort (Pinzke and Lavesson, 2018[51]). The same occurs in other tasks such as "phytosanitary treatments", where most of the postures the worker adopted were with a straight back and walking, obtaining the two lowest risk levels (Table 4(Tab. 4)).

The RULA results for T3 and T5 show the same action level (level 3). One must remember that this method only assesses the most damaging or repetitive posture for each subtask. This is the reason for the high risk level, including for T5 (Table 6(Tab. 6)).

Another task to highlight is harvesting. The four risk levels appear in the two subtasks (T15 and T16). In both, levels 3 and 4 appeared in fewer postures than the lower risks (Figure 4(Fig. 4)). Corrective actions are required for the harmful postures.

The two harvests are carried out in a similar way. The worker must bend down frequently to pick the peppers. The difference is that the method of bending down was usually not the same in the two cases.

**Figure 9:**
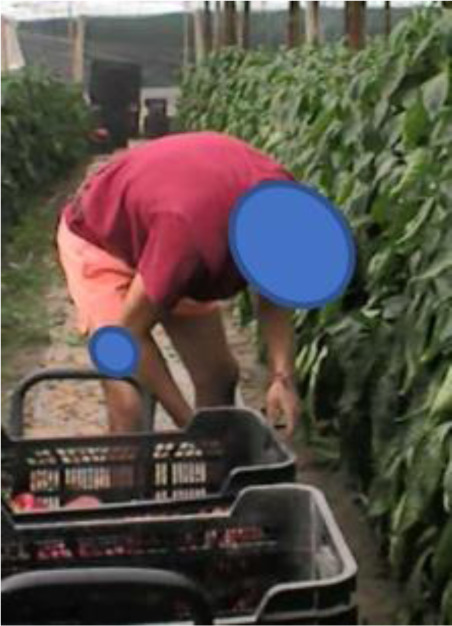
Subtask T15

**Figure 10:**
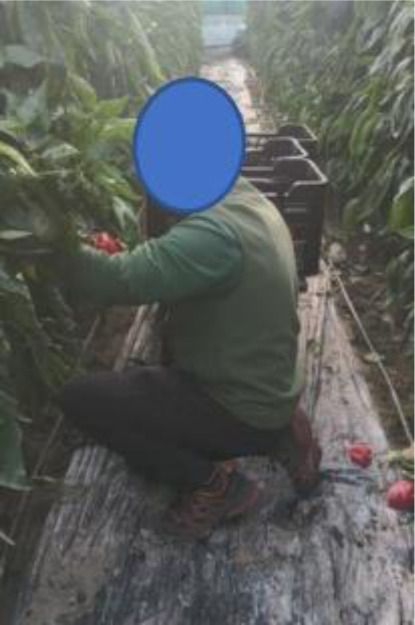
Subtask T16

In the first harvest (T15), the agricultural worker normally crouched down with his back bent and his legs bent. In the other (T16), he would put one knee on the ground. Often, the back could be kept straight with the support of the knee (Table 3(Tab. 3), Figures 9(Fig. 9) and 10(Fig. 10)). In the second case, there were more postures adopted that are considered normal.

Bending over during tasks can lead to musculoskeletal disorders. The results show that the way the posture is performed can partially vary the risk level to which the worker is exposed.

Harvesting is a task that requires forced postures in other crops as well, such as asparagus. Likewise, the workers adopt a crouched position. There are solutions available to avoid this type of posture such as using cutting tools with long handles and collection carts with larger wheels (Sakamoto et al., 2017[55]).

The results according to RULA (Table 6(Tab. 6)) coincide with those of OWAS. They confirm that the first harvest (action level 4) was more harmful than the second (risk level 3). In both subtasks, the way they are performed must be modified, and for the former, this must be done immediately.

In both tasks, the total score was 5 or higher (RULA). One study demonstrated this same range of scores (according to RULA) in the manual harvesting task carried out by agricultural workers (Jain et al., 2018[33]). This task places a high physical demand on the worker, which often leads to MSD. They also showed that the back was affected. These findings agree with the present study. For both harvests (Table 4(Tab. 4)), the bent back acquired a risk level of 2.

Regarding the parts of the body affected during cultivation, the OWAS results showed that some high risks were presented for the bent back (Table 4(Tab. 4)). This posture was adopted in most subtasks. Another greenhouse pepper study agreed that agricultural workers kept their back bent much of the time (Gyemi et al., 2016[25]).

Other authors assessed workers cultivating red pepper, finding MSD in the back, but also in the knees and shoulders (Kim et al., 2009[38]). With OWAS, the study results agree that the legs were also affected by the highest risks (Table 4(Tab. 4)). The shoulders are not assessed in the methods used (OWAS and RULA).

In Iranian agricultural workers, MSDs were determined in the back, knees, and neck. The average RULA score obtained was 6.7 (Dianat et al., 2020[12]). This is a high score, similar to those obtained in our study in most tasks, and which require important changes.

Another study in Shandong greenhouses showed MSD in the same areas (back, knees, shoulders, and neck) as those indicated in the studies cited above (Zheng et al., 2018[65]). Therefore, based on the concurrence among the studies, these parts of the body would often seem to be affected in agricultural tasks.

On the other hand, the load handled throughout the cultivation process is less than 10 kg (Table 4(Tab. 4)). Only when loading the lorry do agricultural workers pick up heavier loads (between 10 and 20 kg). One study demonstrated that the boxes used by agricultural workers to transport greenhouse pepper and tomato should not exceed 12 kg to avoid ergonomic problems (Riemer and Bechar, 2016[53]).

In general, the OWAS results show that, although there are high percentages of postures that are not considered harmful (risk level 1), the number of postures that include some risk is also very high (Figures 4(Fig. 4) and 5(Fig. 5)). Agriculture is a sector in which many workers adopt uncomfortable postures or handle heavy loads (Pardo-Ferreira et al., 2018[49]).

RULA does not indicate the same thing (Figure 6(Fig. 6)) as it presents no posture that is harmful. All the subtasks present risk, with the highest percentages of postures being those with the highest risk. This is justified by the fact that RULA only assesses the most unfavorable postures (McAtamney and Corlett, 1993[43]). It coincides with two studies in which melon cultivation was analyzed. With OWAS, risk levels 3 and 4 had the lowest percentages whereas with RULA, they had the highest (Gómez-Galán et al., 2018[24]; 2019[22]), as was the case in our study.

Both observation methods have a limitation - neither contemplates the duration of the postures. Exposure time is not a factor that is assessed (Takala et al., 2010[60]). Therefore, it has not been considered in this study.

In the pepper cultivation tasks as a whole, there is a group of postures that can be differentiated as repetitive (Table 3(Tab. 3)). The repetitiveness of movements in agricultural work during the work cycle increases the risk of musculoskeletal disorders (Messias and Okuno, 2012[44]). This factor was also indicated in RULA, since one of the criteria that increases the score is that the posture is performed more than 4 times in one minute (INERMAP, 2011[31]; McAtamney and Corlett, 1993[43]). This is true for pepper cultivation and was the reason for selecting postures at small time intervals with OWAS to detect position changes.

 The results obtained with this study are a first step towards research and develop tools that can reduce the physical load. By developing advanced technologies in agriculture, the complexities faced by agricultural workers in carrying out their work would decrease, and productivity would increase (Abrahao et al., 2012[1]).

Although methods such as RULA and OWAS were intended for the industrial sector, they can be applied to agriculture. For this, it will be necessary to adapt to the working conditions present in the sector (Chang, 2011[8]). One of the limitations of this study is that short and often variable time intervals are chosen. This is because agricultural workers change position every few seconds. In addition, they frequently take breaks or carry out maintenance tasks, so consecutive observation is sometimes impossible.

 Another limitation is the subjectivity of the study assessor. The quality of the images is also a determining factor. Some tasks such as "phytosanitary treatments" cannot be recorded from certain positions or distances due to the negative effects of the products used. Therefore, postures are sometimes unclear. In these cases, direct observation plays an important role.

Although OWAS and RULA detect problems in carrying out tasks, this does not limit them being carried out; that is to say, most workers do not request sick leave. This fact might indicate that RULA and OWAS overestimate the risks (Gómez-Galán et al., 2017[23]).

Finally, it is important to adopt measures to avoid possible MSDs in this cultivation. Certain recommendations are presented below:

- Training workers as an effective solution for combating disorders (López-Aragón et al., 2018[41]; Ya'acob et al., 2018[64]). Specific to each pepper cultivation task.
- Developing new technologies that can be applied to this cultivation process (Nwe et al., 2012[48]).
- Using cutting tools with extendable handles (ILO, 2011[29]; Sakamoto et al., 2017[55]). They would be used to harvest the peppers thus avoiding crouching postures.
- Correctly organizing the agricultural workers' labor (López-Aragon et al., 2018[41]).
- Rotating workers between tasks (Barrero et al., 2012[5]). Alternating between subtasks, such as cleaning the greenhouse or preparing the crop.
- Some tasks could be mechanized (ILO, 2011[29]) while seeking a balance with agricultural sustainability (Barneo-Alcántara et al., 2020[4]).
- Encouraging the taking of breaks during each subtask (ILO, 2011[29]).
- Training in lifting pepper boxes. Using forklifts to load the lorry (EU-OSHA, 2012[16]).
- Improving the workers' physical condition (EU-OSHA, 2008[17]).

## Conclusions

In greenhouse pepper cultivation, agricultural workers adopt postures that are detrimental to their musculoskeletal system. Thus, they are continually at risk of developing MSD. In addition, these are repetitive tasks although this does not usually limit them being carried out.

The OWAS and RULA assessment methods allow one to determine the postural risk and activity present in the agricultural sector. Their results do not have to coincide, since RULA assesses the most unfavorable postures while OWAS assesses a group of them. Hence, they are complementary methods.

Measures such a implementing new technologies, modifying tools, training workers, and improving their physical condition can contribute to reducing musculoskeletal risks in pepper cultivation workers.

## Conflicts of interest

The authors declare no conflicts of interest.

## Acknowledgements

We thank the Andalusian Laboratory-Observatory into Working Conditions in the Agricultural Sector (LASA; CG401251) for funding.

## Appendix

### Appendix A

The following table(Tab. 7) (References in Table A1: INERMAP, 2011[31]; Karhu et al., 1977[34]; Takala et al., 2010[60]) presents the meaning of the digits established by the OWAS method for assigning the posture code.

### Appendix B

Table B1(Tab. 8) presents images of the 20 subtasks in pepper cultivation assessed.

### Appendix C:

**Figure 11:**
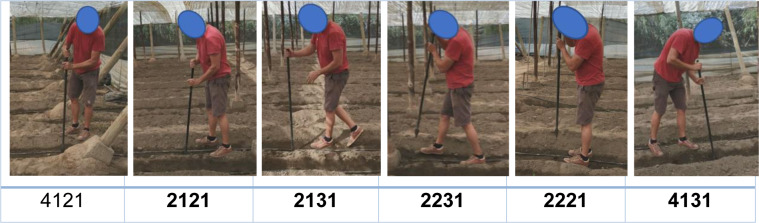
Figure C1: Coding of postures in the “making of holes”, according to OWAS

In the OWAS method, a prior coding of the postures was carried out. The results were obtained from the codes. The following images(Fig. 11) show coding examples for all the different positions in one of the subtasks.

**Figure 12:**
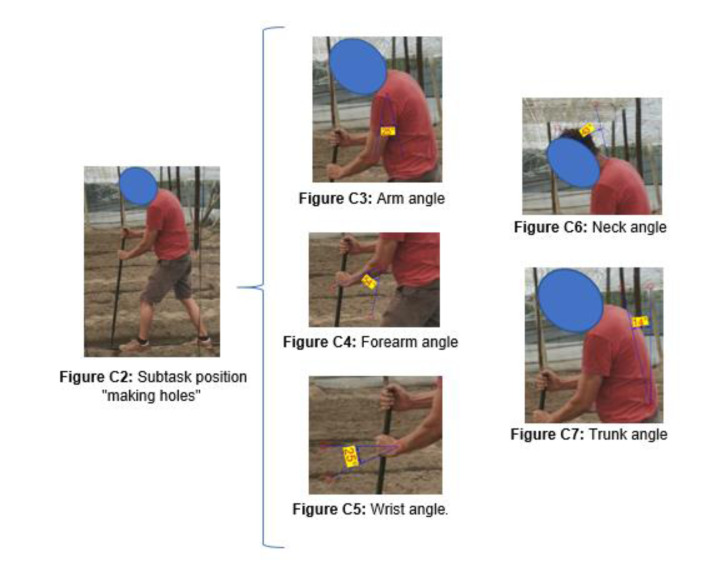
Figures C2-C6: To apply the RULA method, the first step was to measure the angles presented in the selected images. For this, AutoCAD software was used.

To apply the RULA method, the first step was to measure the angles presented in the selected images(Fig. 12).

## Figures and Tables

**Table 1:**
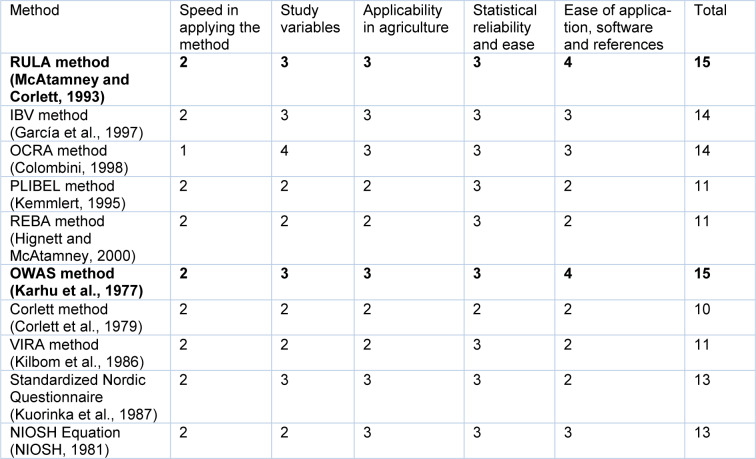
Assessment method decision matrix

**Table 2:**
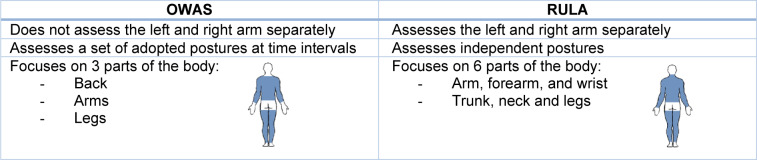
Differences between OWAS and RULA (Karhu et al., 1977; McAtamney and Corlett, 1993; Takala et al., 2010)

**Table 3:**
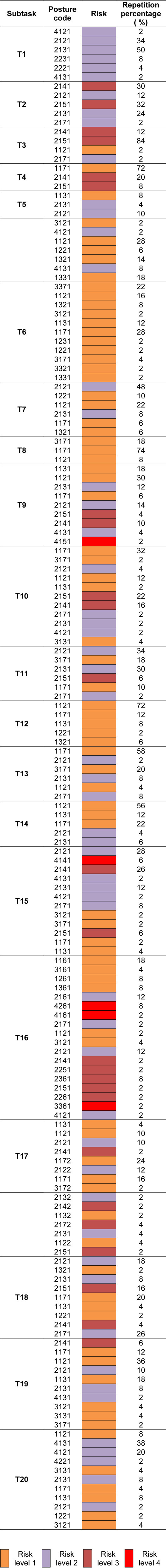
Posture code, risk and repetition percentage

**Table 4:**
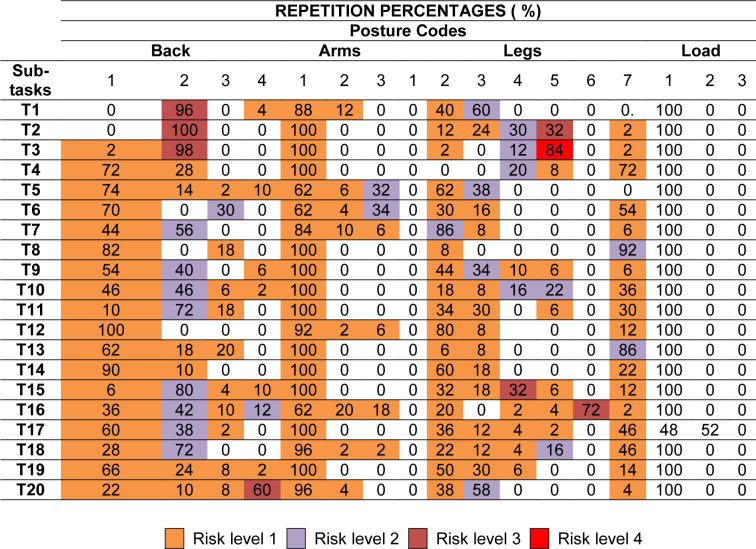
Risk and repetition percentage of each body area

**Table 5:**
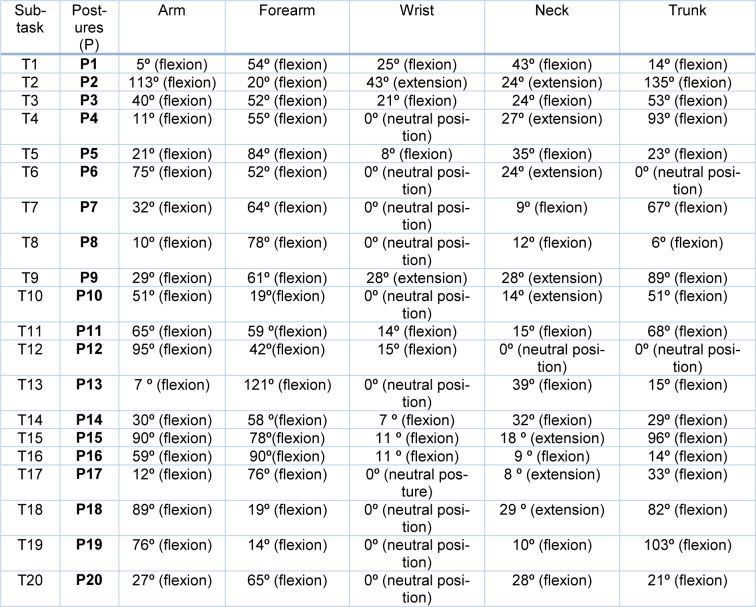
Angles obtained in each part of the body assessed

**Table 6:**
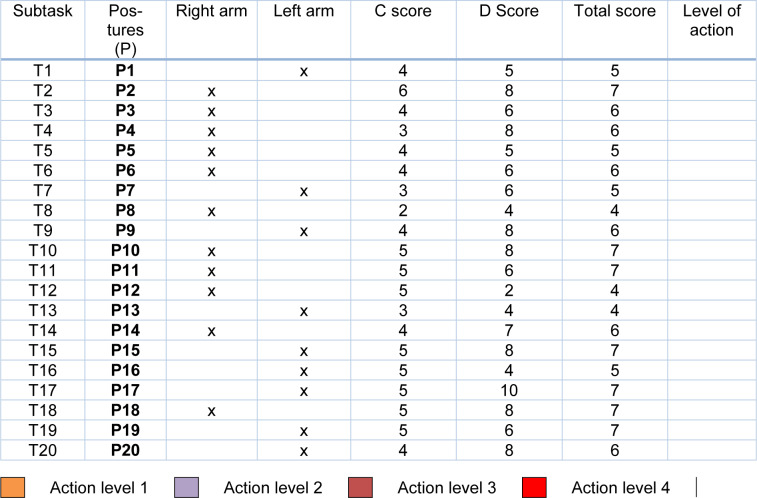
Scores and action levels for each posture

**Table 7:**
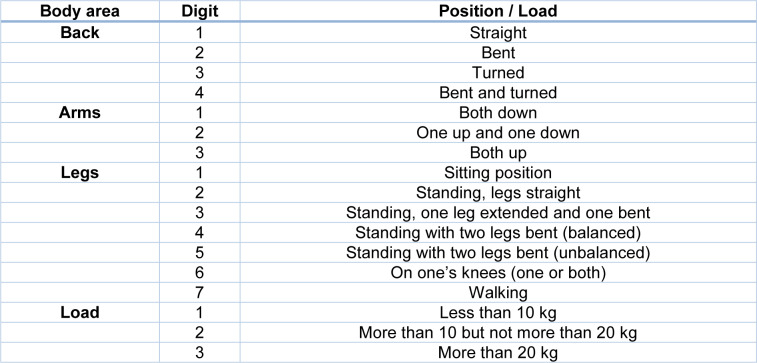
Table A1: Meaning of the digits for the OWAS posture code (INERMAP, 2011; Karhu et al., 1977; Takala et al., 2010)

**Table 8:**
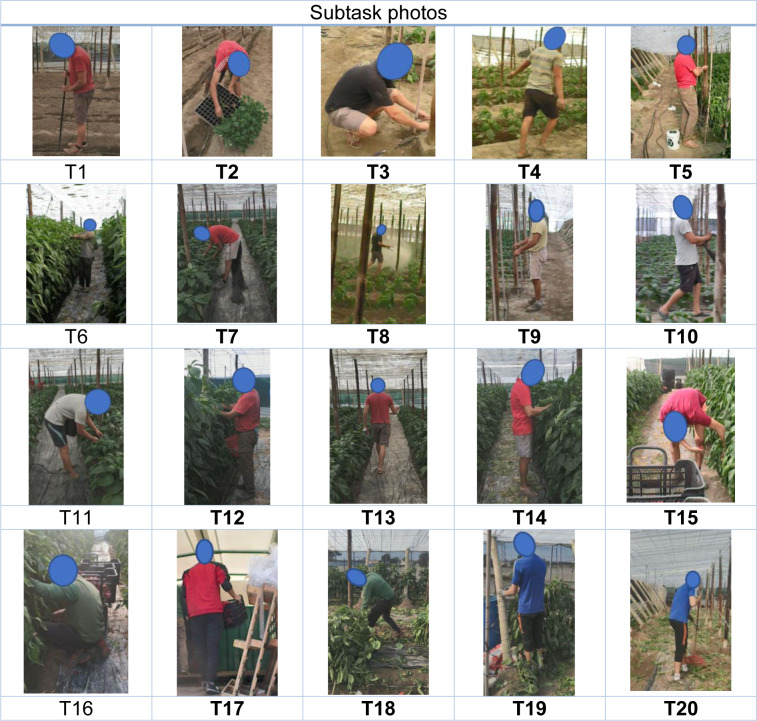
Table B1: Images of the 20 subtasks in pepper cultivation assessed
